# Effects of nephrectomy on respiratory function and quality of life of living donors: a longitudinal study

**DOI:** 10.1590/bjpt-rbf.2014.0105

**Published:** 2015-09-01

**Authors:** Karen Moraes, Denise M. Paisani, Nathália C. T. Pacheco, Luciana D. Chiavegato

**Affiliations:** 1 Programa de Mestrado e Doutorado em Fisioterapia, Universidade Cidade de São Paulo (UNICID), São Paulo, SP, Brazil; 2 Departamento de Fisioterapia, Escola de Medicina, Universidade de São Paulo (USP), São Paulo, SP, Brazil; 3 Disciplina de Pneumologia, Universidade Federal de São Paulo (UNIFESP), São Paulo, SP, Brazil

**Keywords:** kidney living donor, respiratory muscles, postoperative complications, quality of life, lung volume measurements, physical therapy modalities

## Abstract

**BACKGROUND:**

A living donor transplant improves the survival and quality of life of a transplant patient. However, the impact of transplantation on postoperative lung function and respiratory muscular strength in kidney donors remains unknown.

**OBJECTIVE:**

To evaluate pulmonary function, respiratory muscle strength, quality of life and the incidence of postoperative pulmonary complications (PPCs) in kidney donors undergoing nephrectomy.

**METHOD:**

This prospective cohort enrolled 110 consecutive kidney donors undergoing nephrectomy. Subjects underwent pulmonary function (using spirometry) and respiratory muscular strength (using manovacuometry) assessments on the day prior to surgery and 1, 2, 3 and 5 days postoperatively. Quality of life (measured by the SF-36) was evaluated preoperatively and 30 days postoperatively. PPCs were assessed daily by a blinded assessor.

**RESULTS:**

Donors exhibited a decrease of 27% in forced vital capacity, 58% in maximum inspiratory capacity and 51% in maximum expiratory pressure on the 1^st^postoperative day (p<0.001) but this improved over days 2, 3 and 5 but had not returned to preoperative levels. Patient quality of life was still impaired at 30 days with regards to functional capacity, physical role, pain, vitality and social functioning (p<0.05) but these parameters improved slowly. None of the patients developed PPCs.

**CONCLUSION:**

Kidney donors submitted to nephrectomy exhibited a reduction in pulmonary function, respiratory muscular strength and quality of life, most of which were improving toward pre-surgical levels.

## Introduction

The preferred treatment for end-stage chronic kidney disease (CKD) is renal transplantation, which is considered a viable and safe procedure^1^^,^ 2. Living-donor renal transplantation reduces mortality and improves recipients' quality of life with low impact on donors^1^^-^ 4. The donor's risk of developing postoperative complications is inherent to the surgical procedure and is sometimes associated with previous comorbidities^5^^,^ 6.

Donor nephrectomy is considered an upper abdominal surgery (UAS), and several studies have demonstrated that patients undergoing this surgery develop altered respiratory mechanics and pulmonary function, which could lead to pulmonary complications^6^^-^ 8. Studies assessing the evolution of pulmonary function and respiratory muscle strength after UAS reported a 40 to 50% decrease in Forced Vital Capacity (FVC), Forced Expiratory Volume in one second (FEV_1_), and respiratory muscle strength^9^^-^ 13. However, there is relatively little information available on the effects of transplant on pulmonary function in cases of donor nephrectomy.

Postoperative Pulmonary Complications (PPC) are considered one of the major etiologic factors for prolonged hospital stay and mortality, and the incidence rate of PPC varies from 9 to 40% in UAS, depending on the diagnostic criteria used^7^^,^ 14. Chiavegato et al.^5^ reported a low incidence of PPC after assessing two types of nephrectomy incision; however, the patient follow-up period was not sufficient to analyze the clinical repercussions of donation.

The risks for donors are considered low, and Lopes et al.^15^ reported an increase in self-esteem, altruism, and improvement in the quality of life of living donors. However, a small proportion of the donors report negative experiences, mainly after unsuccessful donation, such as transplant rejection and/or postoperative complications^15^^,^ 16. Although donation has a positive global effect on both the donor and the recipient, the true impact of donation on respiratory function as well as on donor quality of life requires further investigation. With this information, new proposals of clinical management could be better applied by multidisciplinary staff. Therefore, this study aimed to assess postoperative pulmonary function and respiratory muscle strength as well as the incidence of pulmonary complications (from 1 to 5 days postoperatively) and the quality of life (at 30 days postoperatively of kidney donors undergoing nephrectomy).

## Method

### Participants

This study is a prospective cohort study that consecutively recruited 124 kidney donors who underwent donor nephrectomy, regardless of the surgical approach (lumbotomy or subcostal), at the *Hospital do Rim and Hipertensão - Fundação Oswaldo Ramos, São Paulo, Brazil*. The *Universidade Federal de São Paulo* (UNIFESP) Ethics Committee, São Paulo, state of São Paulo (SP), Brazil (1127/01), approved this study, and all volunteers provided their signed consent.

### Inclusion and exclusion criteria

We included participants of both genders, over 18 years of age, who underwent donor nephrectomy. We excluded individuals unable to perform the prescribed physical therapy techniques and measurements, previous pulmonary disease, hypertension, diabetes mellitus, and clinically uncontrolled cardiomyopathy, need for postoperative mechanical ventilation for more than 24 hours, death within the first 12 postoperative hours, need for intervention in an organ other than the kidney, and surgical re-intervention.

### Experimental design

All donors were assessed one day before surgery and on the 1^st^, 2^nd^, 3^rd^ and 5^th^ postoperative days. The assessments included anthropometric data, physical examination, pulmonary function (using spirometry), respiratory muscle strength (using manometry) and quality of life (using the SF-36). Chest x-rays were performed in the preoperative period and on the 2^nd^ postoperative day. All donors received preoperative instructions about the surgery and the importance of coughing, walking, and respiratory physical therapy. During the postoperative period, the donors were submitted to physical therapy, which consisted of 15 repetitions of diaphragmatic breathing and another 15 repetitions associated with active mobilization of the upper limbs, in addition to walking under the supervision of a physical therapist every day, from admission until hospital discharge. The physical therapy sessions were performed once a day, with an average duration of 15 minutes per session. Before performing the exercises, pain was assessed using a Visual Analog Scale (VAS)^17^. In cases of pain intensity equal to or greater than five, the donors received medication according to the institution's protocol. Postoperative pulmonary complications were recorded during the first 30 postoperative days. Thirty days after hospital discharge, the donor returned for outpatient assessment of his/her quality of life (SF-36 questionnaire)^18^.

### Assessment methods

#### Pulmonary function:

Spirometry was executed with a portable spirometer, previously calibrated (*Spirobank* ^®^ *, Italy*), using the technical criteria proposed by the American Thoracic Society guidelines for pulmonary function tests^19^.

#### Respiratory muscle strength:

This parameter was assessed by the Maximum Inspiratory Pressure (MIP) and Maximum Expiratory Pressure (MEP), which were obtained by using a manometer (MTR, Brazil), with a scale ranging from 0 to 300 cmH_2_O. Three measurements were taken with the patient seated at 60-second intervals, and the highest value was the recorded score. Both measurements were obtained from functional residual capacity (FRC)^20^. MIP was obtained by asking the patient to breath calmly and then to inhale as much as possible. Simultaneously, the inspiratory limb of the unidirectional valve was occluded. MEP was recorded by asking the patient to breath calmly and then to exhale as much as possible. At this moment, the unidirectional valve was occluded. The mouthpieces used for recording respiratory muscle strength contained a minimal hole, of approximately 2 mm, to exclude facial muscle strength and to increase the reliability of the measurements^20^. The percentage of the predicted values was calculated using the equations proposed by Neder et al.^21^.

#### Postoperative pulmonary complications:

PPCs were characterized by the chest x-ray performed on the 2^nd^ postoperative (PO) day and were analyzed by the lung specialist of the institution based on the PPC diagnostic criteria proposed by Pereira et al.^22^ On the 30^th^ PO day, the presence of PPC was assessed by consulting the medical records.

#### Quality of life:

This parameter was assessed using the SF-36 questionnaire, was given to the subjects by the principal investigator in the preoperative period and on the 30^th^ postoperative day. This validated questionnaire^18^, with a total score of 100 points, included the investigation of 8 domains with 11 questions related to functional capacity, limitations due to physical aspects, pain, general health status, vitality, social aspects, emotional aspects and mental health. Higher scores reflect a better quality of life.

#### Statistical analysis:

We sampled this study by convenience. Descriptive statistics were expressed as mean and standard deviations for continuous variables and as frequencies and percentages for categorical variables. After confirming the normal distribution of the data using the Kolmogorov-Smirnov's test, one-way ANOVA with repeated measures was used to compare the mean values between the preoperative period and the postoperative period (1^st^ and 5^th^postoperatively), and Tukey's *post hoc* test was applied whenever differences were found. Quality of life was analyzed using Student's *t-*tests. A 5% level of significance was adopted for all comparisons. Data were analyzed using the *SigmaStat* 3.2 software (San Jose, USA).

## Results

One hundred twenty-four donors were assessed, 110 (88%) of whom were included in the study; 14 (12%) did not undergo surgery due to issues related to the recipient. The general characteristics of the included patients are presented in Table 1.

**Table 1.** General characteristics of the 110 kidney donors

	Mean±SD
Age (years)	42.21±9.45
Weight (kg)	68.6±13.60
Height (cm)	1.61±0.08
Body mass index (kg/m^2^)	26.23±4.46
Surgical duration (minutes)	105.04±21.24

Kg=kilograms; cm=centimeters; kg/m2=kilograms per square meter; BMI=body mass index; min=minutes.

Among the 110 included donors, 72 (65%) were female, 25 (22.7%) were smokers, 22 (20%) were former smokers, and 8 (7.3%) consumed alcohol. Among the donors, one (0.9%) had been treated for heart failure, one had (0.9%) diabetes mellitus, and nine had (8.2%) hypertension. All patients were stable and controlled (Table 1).

### Pulmonary function

On the pulmonary function assessment, the results revealed a 27% decrease in FVC on the 1^st^ postoperatively (p<0.001) with a gradual recovery; the FVC was decreased by approximately 9% on the 5^th^ PO day when compared with the preoperative values (p>0.05; Table 2).

**Table 2.** Pulmonary function variables preoperatively and on the 1st, 2nd, 3rd and 5th postoperative days for kidney donors (mean±SD).

	PRE	1^st^ PO	2^nd^ PO	3^rd^ PO	5^th^ PO
FVC/ (L)	3.57±0.81	2.64±0.74^*^	2.90±0.76	3.11±0.81	3.24±0.80
% pred	103.19±12.62	76.33±15.93^*^	83.72±4.43	89.71±14.04	93.76±15.57
FEV_1_(L)	2.96±0.83	2.04±0.6^*^	2.27±0.63	2.48±0.69	2.60±0.68^*^
% pred	99.32±12.97	69.76±15.82^*^	76.25±17.67	84.91±15.23	89.17±4.84^*^
FEV_1_/FVC (%)	81.47±7.47	77.17±9.29^*^	78.59±9.39	80.02±8.96	80.68±7.82
% pred	100.03±9.53	94.68±11.72^*^	95.47±14.89	98.37±11.21	98.92±9.94

PRE=preoperative day; PO=postoperative day; FVC=forced vital capacity; L=liters; FEV1=forced expiratory volume in one second; pred=predicted. *P<0.05 comparison with PRE.

When analyzing FEV_1_, a 31% and 12% decrease on the 1^st^ and 5^th^ PO days were observed (p<0.001), respectively, with a gradual return to the preoperative values (Table 2).

The analysis of the FEV1/FVC ratio also revealed a 6% decrease on the 1^st^PO day (p<0.001), returning to values very close to the preoperative values on the 5^th^ PO day (p>0.05; Table 2).

### Respiratory muscle strength

Respiratory muscle strength assessment revealed a 58% decrease in the mean MIP on the 1^st^ PO day (p<0.001; Table 3), with gradual recovery; the values on the 5^th^ PO day were similar to the preoperative ones (p>0.05; Table 3). MEP revealed a 51% and 81% decrease on the 1^st^ and 5^th^ PO days, respectively (p<0.001; Table 3).

**Table 3.** Respiratory muscle strength of kidney donors preoperatively and on the 1st, 2nd, 3rd and 5th postoperative days (mean±SD).

	PRE	1^st^ PO	2^nd^ PO	3^rd^ PO	5^th^ PO
MIP (cmH_2_0)	61.78±18.95	36.15±9.97^*^	41.60±11.69	50.82±16.99	59.62±18.82
% pred	61.52±16.93	36.15±9.14^*^	41.56±10.67	50.37±14.64	59.04±15.77
MEP (cm H_2_0)	71.05±20.73	36.59±12.54^*^	43.65±15.44	49.74±15.25	58.00±18.62^*^
% pred	68.73±17.55	35.53±10.66^*^	42.35±13.20	48.38±13.01	56.26±15.41^*^

PRE=preoperative day; PO=postoperative day; MIP=maximum inspiratory pressure; MEP=maximum expiratory pressure. *P<0.05 comparison with PRE.

### Pulmonary complications and pain

The chest x-ray indicated that 35 (32%) patients exhibited basal lamina atelectasis without clinical change; the findings were thus not considered pulmonary complications.

All patients reported no preoperative pain, with a zero score on the visual analog scale (VAS). On the 1^st^ PO day, the median value of pain was 5 (Min=0 and Max=10; p<0.001), with a gradual decrease in pain over the postoperative period, reaching a median of 1 on the 5^th^ PO day (Min=0 and Max=7; p<0.001).

### Quality of life

When analyzing the 110 donors, differences were observed in most of the domains of the SF-36 questionnaire, when comparing the preoperative and postoperative periods. There was a worsening in the domains of functional capacity, limitations due to physical aspects, pain, vitality and social aspects (p<0.05; Table 4). The values for the domains general health status, emotional aspects and mental health were similar to those observed in the preoperative period (p>0.05; Table 4).

**Table 4.** Quality of life preoperatively and 30 days postoperatively among the 110 kidney donors (mean±SD).

Dimension	Preoperative	Postoperative	P value
Functional capacity	91.09±10.03	77.32±20.53	0.001^*^
Physical role	92.07±19.72	46.22±43.57	0.001^*^
Pain	81.02±17.54	64.29±22.63	0.001^*^
General health	87.71±14.21	90.27±10.87	0.26
Vitality	54.51±12.69	74.63±20.32	0.003^*^
Social function	93.90±11.20	77.74±22.45	0.001^*^
Emotional role	95.94±13.32	89.53±20.05	0.09
Mental health	85.46±11.69	80.29±19.13	0.75

*P<0.05.

## Discussion

This study demonstrated that patients undergoing nephrectomy exhibited decreased pulmonary function and respiratory muscle strength on the 1^st^ PO day compared with the preoperative period, with a gradual return to preoperative values on the 5^th^ PO day; pulmonary complications were not observed. With regards to quality of life, there was no significant difference in the general health status, emotional aspects or mental health; there was, however, a decrease in functional capacity, limitations due to physical aspects, pain, vitality and social aspects at 30 days.

In the present study, less respiratory function impairment in healthy donors who underwent nephrectomy was observed compared with other open upper abdominal surgeries^5^^,^ 7 ^,^ 14 ^,^ 23. The present findings demonstrate that FVC and FEV_1_ decreased by approximately 30% on the 1^st^ PO day; these data differ from those found in other upper abdominal surgeries in which the variables were typically reduced by approximately 50%^24^^-^ 26. In addition, FVC returned to preoperative values on the 5^th^ PO day, which normally occurs seven or more days after surgery^7^^,^ 27. Corroborating these data, Lunardi et al.^27^ observed a 24% decrease in the maximum inspiratory capacity in patients undergoing laparoscopic UAS.

With respect to respiratory muscle strength, a 40% decrease in MIP and a 47% decrease in MEP was observed on the 1^st^ PO day, with a tendency to return to baseline values on the subsequent postoperative days; these data are similar to those previously described in the literature^12^^,^ 26 ^,^ 28. Several possibilities have been suggested in the literature to justify the change in respiratory function and muscle strength during the postoperative period, such as decreasing pain, choice of surgical incision site, duration of anesthesia and surgery, but the primary cause is reported to be diaphragmatic mobility dysfunction due to the reflex inhibition of the phrenic nerve^6^^,^ 7 ^,^ 9. However, continued advances and improvements in the surgical techniques, in addition to the presence of a multidisciplinary team providing care to the patient, seems to contribute to the low pulmonary involvement observed in this study^13^.

Intriguingly, we observed no PPCs; however, 35% of patients exhibited radiological findings that could indicate the presence of, or potential for, such complications. Although the incidence of pulmonary complications in UAS has been reported to range from 9 and 40%^7^^,^ 9, we believe that this variation occurred due to the divergent PPC diagnostic criteria^7^^,^ 9. In the present study, rigorous criteria was used to define PPCs, a fact that might explain the difference in the present findings when compared with previous studies^7^^,^ 10. Interestingly, other studies conducted at the same Institution reported an incidence between 7 and 24% for similar upper abdominal surgery^8^^,^ 29. A possible explanation for this finding is that the present sample consisted of young, healthy participants and that advancements and improvements in preoperative care have occurred throughout the years, including physical therapy, that contribute to PPC prevention^13^^,^ 30.

Although nephrectomy was performed by conventional open surgery, our results are similar to those found in patients undergoing laparoscopic nephrectomy^25^^,^ 26. Studies have suggested that laparoscopic surgery is associated with decreased analgesia, reduced hospital stay, and early return to preoperative conditions^25^^,^ 26. A recent systematic review^25^, however, demonstrated that when comparing laparoscopic and open surgeries, the incidence of general complications such as reoperation, early graft loss and delayed graft function are similar in both procedures. Ferrario et al.^26^ assessed 46 donors undergoing laparoscopic nephrectomy and, as in the present study, found no postoperative complications during the hospital stay; however, although our sample underwent open nephrectomy, shorter durations of surgery (105.04±21 vs. 170±45 minutes) and of hospital stay (3 vs. 5±1 days) were observed.

When analyzing quality of life, the present study showed differences in functional capacity, limitations due to physical aspects, pain, vitality and social aspects following surgery, corroborating the previous findings of other authors^15^^,^ 16 of the negative impact of donation on the physical and social characteristics of patients, mostly women in the same age group as the present study (mean age between 40 and 50 years). However, there were no changes in general health status, emotional aspects or mental health, and this fact could mean that personal and emotional satisfaction for organ donation is truly present^15^. Although donors exhibit changes in pain and physical and functional capacity, studies suggest that donors return to pre-donation levels of daily living activities within a short time^15^^,^ 16; however, there is no consensus on when exactly this occurs. Although the present study assessed quality of life 30 days after donation, changes in certain domains might have been due to the surgery itself, similar to any other procedure of this magnitude.

An important difference in the present study was that all patients underwent respiratory physical therapy once a day in the pre- and postoperative periods. Information regarding the surgical incision, the importance of coughing, early walking and physical therapy exercises was provided during the preoperative period. In the postoperative period, respiratory exercises were performed under supervision. Because the study was performed in a university hospital with growing care difficulties and poor pre- and postoperative care, the authors expected to observe greater repercussions on pulmonary function and strength associated with postoperative complications. However, unlike other surgical patients, the present sample consisted of healthy participants who had a previous awareness of the importance of physical therapy and the fact that physical therapy was performed under supervision might explain the present findings.

This study has some limitations. First, all patients underwent physical therapy postoperatively, and because there was no control group, the authors could not determine whether physical therapy contributed to the favorable outcomes observed in the present study. Second, the chest x-rays used for PPC assessment were analyzed by a single lung specialist. Third, quality of life was assessed at 30 days following the institutional practice routine; however, the authors believe a longer follow-up period is required to observe if the quality of life improves over time.

## Conclusion

The results of the present study suggest that donors undergoing nephrectomy and under physical therapy care exhibit reduced pulmonary function and respiratory muscle strength on the 1^st^ PO day, with gradual return to preoperative values by the 5^th^ PO day, and do not suffer pulmonary complications. At 30 days, the quality of life reflects that patients still did not returned to preoperative levels. This happened probably due to the functional capacity and limitations due to physical aspects, pain, vitality and social aspects. However, general health status, emotional aspects and mental health remain unchanged.

## References

[1] Vanbelleghem H, Vanholder R, Levin NW, Becker G, Craig JC, Ito S (2007). The kidney disease: improving Global Outcomes website: comparison of
guidelines as a tool for harmonization. Kidney Int..

[2] Medina-Pestana JO (2006). Organization of a high-volume kidney transplant program--the "assembly
line" approach. Transplantation.

[3] Perlis N, Connelly M, D'A Honey JR, Pace KT, Stewart R (2013). Evaluating potential live-renal donors: causes for rejection, deferral
and planned procedure type, a single-centre experience. Can Urol Assoc J..

[4] Ibrahim HN, Foley R, Tan L, Rogers T, Bailey RF, Guo H (2009). Long-term consequences of kidney donation. N Engl J Med..

[5] Chiavegato L, Medina-Pestana J, Tedesco-Silva H, Paisani D, Fiore J, Faresin S (2010). Surgical approach does not affect perioperative respiratory morbidity
in living donor nephrectomy: comparison between anterior subcostal incision and
flank incision. Transplant Proc.

[6] Smetana GW, Lawrence VA, Cornell JE, American College of Physicians (2006). Preoperative pulmonary risk stratification for noncardiothoracic
surgery: systematic review for the American College of Physicians. Ann Intern Med.

[7] Smetana GW (2009). Postoperative pulmonary complications: an update on risk assessment
and reduction. Cleve Clin J Med.

[8] Paisani DM, Fiore JF, Lunardi AC, Colluci DBB, Santoro IL, Carvalho CRF (2012). Preoperative 6-min walking distance does not predict pulmonary
complications in upper abdominal surgery. Respirology.

[9] Scholes RL, Browning L, Sztendur EM, Denehy L (2009). Duration of anaesthesia, type of surgery, respiratory co-morbidity,
predicted VO2max and smoking predict postoperative pulmonary complications after
upper abdominal surgery: an observational study. Aust J Physiother.

[10] Arozullah AM, Conde MV, Lawrence VA (2003). Preoperative evaluation for postoperative pulmonary
complications. Med Clin North Am..

[11] Chetta A, Tzani P, Marangio E, Carbognani P, Bobbio A, Olivieri D (2006). Respiratory effects of surgery and pulmonary function testing in the
preoperative evaluation. Acta Biomed.

[12] Barbalho-Moulim MC, Miguel GP, Forti EM, Campos FA, Costa D (2011). Effects of preoperative inspiratory muscle training in obese women
undergoing open bariatric surgery: respiratory muscle strength, lung volumes, and
diaphragmatic excursion. Clinics (Sao Paulo).

[13] Grams ST, Ono LM, Noronha MA, Schivinski CI, Paulin E (2012). Breathing exercises in upper abdominal surgery: a systematic review
and meta-analysis. Rev Bras Fisioter..

[14] Arozullah AM, Daley J, Henderson WG, Khuri SF (2000). The National Veterans Administration Surgical Quality Improvement
Program. Multifactorial risk index for predicting postoperative respiratory
failure in men after major noncardiac surgery. Ann Surg.

[15] Lopes A, Frade IC, Teixeira L, Almeida M, Dias L, Henriques AC (2013). Quality of life assessment in a living donor kidney transplantation
program: evaluation of recipients and donors. Transplant Proc.

[16] de Groot IB, Schipper K, van Dijk S, van der Boog PJ, Stiggelbout AM, Baranski AG (2012). Decision making around living and deceased donor kidney
transplantation: a qualitative study exploring the importance of expected
relationship changes. BMC Nephrol.

[17] Bodian CA, Freedman G, Hossain S, Eisenkraft JB, Beilin Y (2001). The visual analog scale for pain: clinical significance in
postoperative patients. Anesthesiology.

[18] Ciconelli RM, Ferraz MB, Santos W, Meinão I, Quaresma MR. (1999). Tradução para a língua portuguesa e validação do questionário genérico
de avaliação de qualidade de vida SF-36 (Brasil SF-36). Rev Bras Reumatol..

[19] American Thoracic Society (1995). Standardization of Spirometry, 1994 Update. Am J Respir Crit Care Med..

[20] Souza R (2002). Maximum static respiratory pressure. J Pneumol..

[21] Neder JA, Andreoni S, Lerario MC, Nery LE. (1999). Reference values for lung function tests. II. Maximal respiratory
pressures and voluntary ventilation. Braz J Med Biol Res..

[22] Pereira ED, Fernandes AL, Anção MS, Pereres CA, Atallah AN, Faresin SM (1999). Prospective assessment of the risk of postoperative pulmonary
complications in patients submitted to upper abdominal surgery. Sao Paulo Med J..

[23] Damiani G, Pinnarelli L, Sammarco A, Sommella L, Francucci M, Ricciardi W (2008). Postoperative pulmonary function in open versus laparoscopic
cholecystectomy: a meta-analysis of the Tiffenau index. Dig Surg..

[24] Paisani DM, Chiavegato DL, Faresin SM (2005). Lung volumes, lung capacities and respiratory muscle strength
following gastroplasty. J Bras Pneumol.

[25] Wilson CH, Sanni A, Rix DA, Soomro NA (2011). Laparoscopic versus open nephrectomy for live kidney
donors. Cochrane Database Syst Rev.

[26] Ferrario M, Buckel E, Astorga C, Godoy J, Aguiló J, González G (2013). Results in laparoscopic living donor nephrectomy: a multicentric
experience. Transplant Proc.

[27] Lunardi AC, Paisani DM, Tanaka C, Carvalho CR (2013). Impact of laparoscopic surgery on thoracoabdominal mechanics and
inspiratory muscular activity. Respir Physiol Neurobiol.

[28] Lunardi AC, Miranda CS, Silva KM, Cecconello I, Carvalho CR (2012). Weakness of expiratory muscles and pulmonary complications in
malnourished patients undergoing upper abdominal surgery. Respirology.

[29] Filardo FA, Faresin SM, Fernandes ALG (2002). Index for a pulmonary postoperative complication after upper abdominal
surgery: a validation study. Rev Assoc Med Bras.

[30] Lawrence VA, Cornell JE, Smetana GW, American College of Physicians (2006). Strategies to reduce postoperative pulmonary complications after
noncardiothoracic surgery: systematic review for the American College of
Physicians. Ann Intern Med.

